# Precision feeding and precision nutrition: a paradigm shift in broiler feed formulation?

**DOI:** 10.5713/ab.21.0034

**Published:** 2021-02-14

**Authors:** Amy F. Moss, Peter V. Chrystal, David J. Cadogan, Stuart J. Wilkinson, Tamsyn M. Crowley, Mingan Choct

**Affiliations:** 1 School of Environmental and Rural Science, University of New England, Armidale, 2350, NSW, Australia; 2 Baiada Poultry Pty Limited, Pendle Hill, 2145, NSW, Australia; 3 Feedworks Pty Ltd, Romsey, 3434, Vic, Australia; 4 Poultry Hub Australia, University of New England, Armidale, 2350, NSW, Australia; 5 School of Medicine, Deakin University, Geelong, 3217, VIC, Australia

**Keywords:** Phase Feeding, Feed Delivery, Digestible Lysine, Energy, Poultry

## Abstract

Broiler chickens grow rapidly, and their nutrient requirements change daily. However, broilers are fed three to five diet phases, meaning nutrients are under or over-supplied throughout production. Increasing diet phases improves production efficiency as there is less time in the production cycle that nutrients are in under or over-supply. Nevertheless, the process of administering four or more diets is costly and often impractical. New technologies are now available to blend feed to match the daily nutrient requirements of broilers. Thus, the aim of this review is to evaluate previous studies measuring the impact of increasing feed phases on nutrient utilisation and growth performance, and review recent studies taking this concept to the extreme; precision nutrition - feeding a new diet for each day of the production cycle. This review will also discuss how modern precision feeding technologies have been utilised and the potential that new technologies may bring to the poultry industry. The development of a precision nutrition regime which targets daily requirements by blending dietary components on farm is anticipated to improve the efficiency of production, reduce production cost and therefore improve sustainability of the industry. There is also potential for precision feeding technology along with precision nutrition strategies to deliver a plethora of other management and economic benefits. These include increased fluidity to cope with sudden environmental or market changes, and the ability to alter diets on a farm by farm level in a large, integrated operation. Thus, the future possibilities and practical implications for such technologies to generate a paradigm shift in feed formulation within the poultry industry to meet the rising demand for animal protein is also discussed.

## INTRODUCTION

Poultry production is one of the largest sources of animal protein supply for human consumption in the world. For many countries, like Australia, chicken-meat is the dominant animal protein, therefore production must continue to expand to supply increasing demand. In Australia, for instance, per capita consumption of chicken-meat is predicted to increase by 7.9% over the next 5 years [1]. Furthermore, feed represents 65% of total chicken-meat production cost [2], and thus improvements in the efficiency of chicken-meat production will ensure the industry can meet rising demand and deliver an affordable product, despite challenges such as increasing wheat prices during drought conditions [3]. Aside from generating economic gains, improving the efficiency of chicken-meat production also reduces the burden on environmental systems. For example, in some cases the pressure to clear land for feed crops is threatening biodiversity [4], and any nutrients that are not absorbed by the animal may contribute to environmental pollution via water run-off from discarded poultry manure [5]. However, reducing excess nutrient in the diet can dramatically reduce the capacity for environmental pollution. For instance, reducing crude protein content in broiler diets by less than 2 percentage units was reported to reduce litter N content by 18% [6]. Hence, poultry nutrition is an opportune area to improve efficiency and enhance the capacity, profitability and sustainability of the Australian chicken-meat industry. Therefore, a large amount of focus is placed on improvement of the utilisation of dietary nutrients. One such way to enhance the utilisation of energy and amino acids is by reducing the amounts that are in shortage or excess throughout the production cycle by optimally meeting nutrient requirements consistently throughout production.

**Figure 1:**
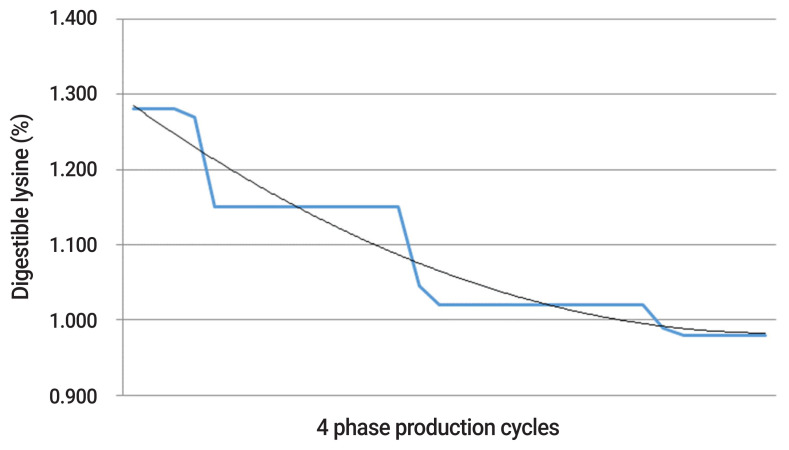
The over and under-supply of dietary nutrient between predicted nutrient requirement (thin black line) and supplied nutrient (thick blue line) as demonstrated for digestible lysine level (%) over a 4-phase production cycle.

**Figure 2:**
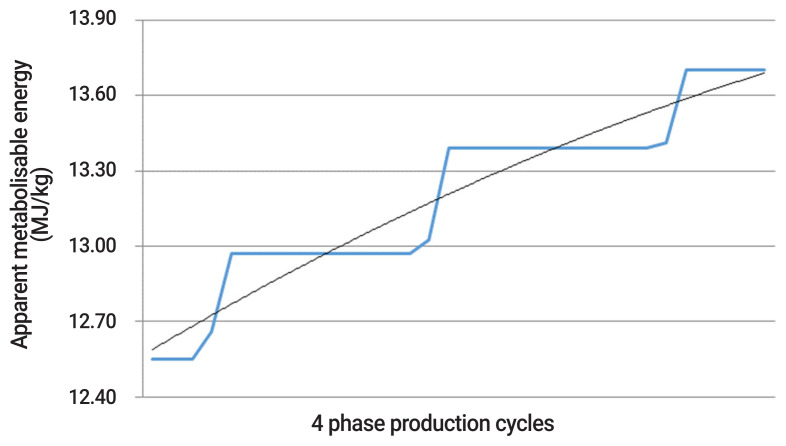
The over and under-supply of dietary nutrient between predicted nutrient requirement (thin black line) and supplied nutrient (thick blue line) as demonstrated for apparent metabolisable energy (MJ/kg) over a 4-phase production cycle.

Modern broiler chickens grow rapidly and as such, their nutrient requirements change daily throughout their production [7]. However, broilers are fed between three and five diets or phases in commercial practice, meaning nutrients are often under and over supplied throughout production. For example, the predicted nutrient requirement and supplied nutrient requirement are demonstrated for digestible lysine and energy over a four phase production cycle in Figures 1 and 2. It is evident that the supply of digestible lysine and energy is in disparity with their requirement over the majority of production. It is also noteworthy that the magnitude of over and under supply of digestible lysine and energy is greatest within the first half of the production cycle, a time which is critical for development and growth. With the nutrient requirement not precisely met throughout the majority of production, a depression in growth (in the case of under supply) or inefficient use of nutrient (in the case of over supply) is probable, and costly for industry. When dietary intake of nutrients is high in comparison to the requirement, excess energy may be stored as fat, and amino acids may be deaminated [8]. This deamination of amino acids is an energy expensive process and leads to ammonia formation, with the majority of ammonia excreted as uric acid [9]. Additionally, microbes within the hind gut of poultry may also utilise excess amino acids to synthesise microbial proteins [10], ammonia and amines [11] and use the excess protein as an energy source [12]. Largely unavailable to poultry due to their location within the hind gut, these microbial proteins and ammonia are excreted and thereby contribute to pollution of the environment. Reduction in nutrient utilisation may also encourage the growth of pathogenic bacteria in the gastrointestinal tract [13], which can be a contributing factor to predispose broilers to *Clostridium perfringens* and necrotic enteritis [14]. Therefore, not only are these energy expensive processes within the bird to compensate for under and over supply of nutrients, but it is a costly and inefficient use of nutrients for the poultry industry.

To minimise the disparity between nutrient supply and nutrient requirements, more precise formulation of diets is required. This may be achieved by increasing the number of feed phases to create a more dynamic adjustment of nutrient supply, even to the extent of meeting daily requirements. Precision nutrition is the practice of adjusting and feeding diets to ensure the dietary nutrient supply equals the nutrient requirement of the animals to a daily basis [15]. This essentially removes the under and over-feeding which is apparent in standard phase-feeding systems consisting of three to five diets (Figures 1 and 2).

Precision nutrition strategies which involve pelleting and feeding a new diet each day to precisely match requirements have been inaccessible to the poultry industry as a large amount of automation, accuracy in feed consumption data, feed storage and feed transport is required, which is simply not practical. However, new and emerging feed weighing and blending technologies—called precision feeding technology—may allow the implementation of such programs by industry without requiring extra investment in feed pelleting, transportation or storage. Precision feeding is the ability to precisely measure the amount of feed delivered to chickens, and hence accurately calculate feed intake and feed conversion ratio. Many of these technologies are equipped with the capacity to blend multiple feeds, and hence allow the development of precision nutrition regimes for poultry production.

Therefore, the aim of this review is to discuss the potential use of precision feeding technology within industry, evaluate previous studies measuring the impact of increasing feed phases on nutrient utilisation and growth performance, and review recent studies taking this concept to the extreme; precision nutrition - feeding a new diet for each day of the production cycle. Finally, this review discusses the potential that new technologies may bring to the poultry industry to optimise production, enhance our control and enable the precision feeding of poultry in the Australian chicken-meat industry to achieve better economic, environmental and social outcomes.

## PRECISION NUTRITION

Successful implementation of precision nutrition for poultry involves meeting three key requirements. Firstly, accurate ingredient characterisation is key to ensure the finished feed represents the intended formulated diets. This is difficult when the nutrient specifications of feed ingredients are highly variable due to varying management, breed, year of production and environmental conditions [16]. Consequently, near-infra red calibrations used by integrated operations instantaneously estimate the nutrient composition of feedstuffs; however, many nutritionists still refer to book values. Thus, understanding the potential amount of variation within the data presented is extremely important instead of just relying on mean values. One important measure to determine the accuracy of the data, and the potential variability that may exist within an ingredient, is the standard deviation. Databases such as the Australian Feed Ingredient Database [17] compile data from multiple source companies and by providing the standard deviation of the data, allow nutritionists to gauge the uncertainty that exists within the data. This information on the potential variability in the ingredient can be fed into stochastic feed formulation programs to formulate diets to the particular level of certainty (or probability) the nutritionist is comfortable with, providing a safer way to calculate safety margins.

Secondly, it is imperative that the nutrient requirements of broilers are accurately determined in order to identify the daily requirement. Enormous amounts of study have been dedicated to pinpointing the requirements of nutrients for broilers. This data may be fed into more sophisticated modelling tools such as the EFG broiler model [18], or may be more simply used by fitting curves to the identified nutrient specification over the starter, grower, finisher and withdrawal phases in order to identify the daily requirement. It stands to reason that the more data points known the better the fit of the regression will be and hence the more successful the precision feeding regime; but it is likely that all the information required already exists in order to generate accurate daily nutrient requirement data.

Thirdly, precision nutrition requires careful management to ensure the prior two requirements are met as closely as possible and to design the feeding regime to meet the daily needs of broilers. This approach provides increased flexibility in that dietary changes may be implemented throughout the production cycle if required, thus aiding interventions, which may be required during production as, discussed in ‘further practical considerations’.

Precision nutrition is not a new concept. The first research to lay the foundation for the precision nutrition approach was done many years ago and explored the use of increasing diet phases beyond one to three diets throughout the production cycle. The concept of increasing diet numbers or phases to improve production efficiency and to reduce the time in the production cycle that nutrients are in under or over supply has been demonstrated, just not to the scale of a diet for every day of production [19–21]. For example, Warren and Emmert [21] compared broilers offered a three phase regime to those offered a single diet based on NRC recommendations between 40 to 61 days. Feeding broilers on the three phase regime improved gain to digestible lysine intake by 6.5% (50.9 vs 54.2; p<0.05) and subsequently reduced feed cost per bird. Kleyn [20] also presented a cost comparison which demonstrates that a three phase diet reduces feed cost by 3.72% compared to a two phase diet as nutrients are used more efficiently.

Sharma et al [22] effectively compared a two phase regime with a 10 phase regime by offering broilers a nutrient dense starter diet that was then subsequently diluted with whole wheat in increasing increments every four days up to 40 days post-hatch. The broilers offered the diets diluted with whole wheat did not exhibit a significantly different weight gain or carcass composition than broilers offered standard starter and grower phases. Feed conversion ratio was compromised in birds offered the blended diets; however, this study did not balance the whole wheat dilution with the bird’s nutrient requirement – a design flaw which may explain the compromised efficiency. Thus, careful consideration needs to be taken to ensure the bird’s requirement is estimated correctly for all diets when the number of dietary phases is increased. Hauschild et al [19] blended two feeds in order to create a 14 phase feeding program over 1 to 42 days of production. Implementation of the 14 phase program increased daily weight gain by 2.1% and breast meat yield by 3.1% while not significantly influencing feed conversion in comparison to a standard four phase feeding program. Therefore, the concept of increasing dietary phases by blending rations to meet the daily energy and lysine requirements may improve production and reduce feed costs when the diets are adequately balanced to requirements.

Increasing the number of feed phases increases bird efficiency, a fact the industry has been aware of for some time. However, the process (pelleting, transporting, and storing) of administering four or more diet phases in practice is expensive, logistically challenging and is often impractical. Thus, integrated broiler operations are often limited to starter, grower, finisher and withdrawal diets. At times, a pre-starter or second withdrawal diet may be implemented, but this is often limited by on-farm storage constraints, mill capabilities and logistics. But, with advancing technology and computer science, new technologies are now available that may facilitate the use of more diets and even allow for the changing of the diet daily to better meet the birds’ requirements and be potentially more cost-effective.

Precision nutrition involves the adjustment of diets offered broilers to ensure the dietary nutrient supply equals the daily nutrient requirement, and thus takes the original multi-phase feeding concept to the extreme. Tailoring feed to the daily nutrient requirement of the flock may be done by predicting intake and growth, either via broiler growth models such as the EFG broiler model [18] or fitting curves to the current breed nutrient specification as done in Figures 1 and 2. From these curves, an energy dense and a protein dense dietary component may then be blended using modern feed delivery systems to precisely meet the predicted nutrient requirements on a daily basis.

Modern feed delivery systems previously discussed may be installed on farm and programmed to automatically blend a protein-dense concentrate that can be subsequently diluted with a low protein but energy-dense concentrate on a daily basis. In this way, two pelleted concentrates may be combined to create a new diet daily in order to achieve the optimal digestible lysine to energy intake across the entire production cycle. As only two dietary components are used in the process, the profitability of this regime would not be hindered by the practicalities of pelleting multiple different diets, feed transportation and storage. Thus, with this new technology, it may be possible to tailor the nutrient profile to the daily requirement to optimise production efficiency.

The precision nutrition concept for broilers was recently explored by Feedworks using the ‘Feed Logic’ [23] precision feeding system to blend a protein dense and an energy dense dietary component to meet daily nutrient requirements [16]. The dietary components were pelleted separately which the Feed Logic precision feeding technology blended these components together in the appropriate proportions to achieve a diet of the desired energy and protein level. The trial comprised a standard three phase feeding program which was compared to three precision feeding programs, each of which had the daily nutrient requirements of the broiler estimated using different methodology, either; the Arbour Acres nutrient requirements, EFG broiler growth model [18], or adjusting the diet blends as the trial progressed to suit the current live weight (subsample measured every two days). Over the 42 day trial, there was no significant difference in growth performance. However, all precision nutrition treatments significantly increased dressing weight and dressing percentage. This study confirms results observed in previous studies examining the effect of feeding greater numbers of diets; feed efficiency itself is not necessarily impacted but nutrient utilisation is improved, as demonstrated in the Warren and Emmert [21] study. Thus, it is sensible that if amino acids are being deposited more efficiently to muscle rather than being deaminated, and less energy is being converted to fat, a more favourable body composition will be developed where dressed weights and dressing percentage are improved. Weight gain or final live weight itself need not necessarily be improved as the improvement appears to be mostly generated in changes in the carcass composition. This is an important distinction as the end product is essentially muscle mass, not live weight.

As a consequence of the improved dressed weight, calculated wholesale returns were approximately $1.30 per bird greater than the standard three phase feeding program. If this improvement can be also observed in commercial practice, this would generate an extra $32,500 of income per flock of 25,000 birds, and should justify the initial cost of the feeding system required. Nevertheless, the aforementioned study is the only study to implement precision nutrition on a daily basis over their entire production cycle, and thus further research is certainly warranted.

The implementation of precision nutrition relies on the ability of the poultry industry to employ precision feeding within their operations. Implementing such nutritional programs may in fact be more achievable than at first glance. Some broiler sheds may already be equipped with feed weighing systems which may be used to implement this concept. For example, many feed weighing systems that already exist within broiler sheds are organised such that 2 or 3 silos each have an auger which connects to a hopper on the input side of the scale. Thus, if the augers may be calibrated to deliver a certain percentage of feed, simple feed weighing equipment may be used to blend either; a protein and energy concentrate or even blend starter, grower and finisher diets, for example, to meet daily lysine and energy requirements and thereby achieve the precision nutrition concept.

## PRECISION FEEDING

The concept of precision agriculture has rapidly expanded since the development of technologies such as remote sensing and automated control, and has been applied to many agricultural systems dramatically reducing cost, increasing yield and leading to more sustainable agriculture [24]. Previously, this technology has only been applied to animals requiring larger investment as the cost for the initial installation of these systems has been expensive. Due to the large proportionate cost of feeding larger animals within an intensive production system, the concept of using precision feeding technology to precisely feed out and monitor intake of diets has been employed to accurately monitor the performance of animals and reduce feed wastage.

To deliver the precision nutrition concept within the poultry industry, precision feeding technology is required, as well as the expertise to utilise the technology and the logistics of implementing and installing the technology into poultry sheds. The logistics of installing precision feeding technology and expertise to run such technology within poultry farms may be more plausible than initial expectations. Currently, some Australian poultry producers, for example, have silos equipped with weigh scales, feed weighing systems or similar equipment to measure the amount of feed delivered to poultry and measure the amount of feed remaining within or at the end of a production phase. However, many still solely rely on estimating the amount dispatched to a given poultry shed. This rough estimation makes monitoring production more difficult for the grower and can lead to performance data being accidentally skewed. For example leftover feed from the previous cycle may be incorporated into the next cycle to minimise feed waste, but may lead to inaccurate feed efficiency calculations for both cycles of birds. Precision feeding equipment entails the use of systems which record the amount of feed being fed out within a shed. Typically, these systems intake feed from a silo and then use a weigh scale, augers calibrated so that each step of the motor dispatches a given amount of feed, or both, to weigh the amount of feed dispatched into the feed lines. Examples of such equipment are the AIRFEED [25] or the Electronic Feed Weigher [26] systems which are already employed into poultry farms and piggeries around the globe. Importantly, these systems also have the capacity to intake feed from more than one auger and blend the feed to chosen ratios. This functionality is unfortunately seldom used, but may be the key to implementing precision nutrition within poultry production. For example, with only two silos connected to such a feeding system, it may be possible to blend a protein and an energy concentrate together to precisely achieve the daily protein and energy requirements. Thus, the use of technology—which in some cases already exists on farm—to employ precision feeding of diets may help the industry to more accurately monitor performance, improve poultry management and decision making, and gives the producer the ability to employ precision nutrition. Previously, this sort of technology was out of producer’s reach due to high cost; however, with the decreasing cost of technology, precision feeding systems are now becoming more common within poultry production. The continuing growth in the capacity for emerging technology in the precision agriculture field may allow the implementation of precision nutrition, and thus this strategy warrants further consideration for implementation to poultry production.

Precision feeding technology has been implemented in piggeries to realise precision nutrition and is an effective way to reduce the amount of nutrient excreted into the environment without compromising growth performance or carcass composition [27]. Precision feeding also allows diets to be adjusted in real time to reflect the pig’s intake and growth [27,28]. Such fine diet control would be particularly effective in broiler production systems due to their extremely rapid growth. These technologies may also generate additional management benefits including easy application of treatments to the flock, automating feed supply and records of feed intake, and reducing ammonia emissions [15], which are also key considerations for broiler production. Precision feeding technologies in piggeries have also allowed real-time prediction and forecasting of future requirements of the pigs throughout their growth via the use of modelling software [29], which may also advantage management of large intensive production systems. With the cost of this technology falling, precision feeding systems are now becoming a possibility for mainstream chicken-meat production. Within the poultry industry, precision feeding technology has been previously implemented in research-based scenarios, albeit sparsely, to assess broiler feed intake and feeding behaviour via feeding stations equipped with electronic balances [30]. However, more recently, precision feeding systems have been employed to manage the feed intake of broiler breeders.

Broiler breeders were offered amounts of feed matched to their daily intake allocation (but not balanced to their daily nutrient requirement) via precision feeding feed stations in Zuidhof et al [31]. Maintaining and restricting daily feed intake is extremely important within breeder production to promote their longevity and fertility. Precision feeding increased feed efficiency by 4.6%, equating to a potential feed saving of >$60 million, in Australian industry terms, by sparing the production of 145,000 tonnes of feed. Additionally, precision feeding of broiler breeders was demonstrated to reduce variation in flock uniformity to less than 2% [32], and may present bird welfare advantages as skip-a-day feeding can be avoided [33]. This control of daily intake was achieved using a precision feeding system developed at the University of Alberta [34] which tracks, weighs and feeds individual broilers based on their current body weight versus their target weight. The advantage of this system is complete control of feed delivered and data for the weight of every individual bird within a flock rather than only flock data. However, with this high level of precision comes an expensive initial cost of the equipment. Nevertheless, in a more expensive bird such as the broiler breeder, ensuring reproductive success with consistent flock uniformity may be worth the initial outlay and the return on investment will continue to improve as technology becomes more affordable.

Zuidhof’s work [31] demonstrates the improvement in performance and control of flock growth that may be achieved when precision feeding technology is incorporated into the breeder management system. However, this is achieved by managing intake in order to precisely control a breeder’s growth, and therefore does not alter the nutrient content of the feed. This system also operates on the individual broiler level. In the utilisation of this concept for broilers, working on a whole-flock level, a different approach may be required.

## FURTHER PRACTICAL CONSIDERATIONS

Precision nutrition requires a well-characterised nutrient database together with a set of properly defined nutrient requirements, which may require some investment to achieve the accuracy and capacity required. However, its implementation may lead to a paradigm shift in poultry feed formulation to provide the increased capacity required to meet the rising demand for chicken-meat. The establishment of precision feeding systems within industry will likely improve efficiency of lean meat production and thus reduce diet costs per kilo carcass weight as previously discussed, improving the economic sustainability of the industry. However, further benefits beyond those reported in the literature may also be advantageous to the poultry industry and therefore warrants discussion. Due to the daily blending of feed from two components, the capacity for diets to be progressively adjusted to reflect the performance of the flock over time, or to cope with a sudden change in conditions at the poultry facility, may also bring benefits. For example, if two dietary components are blended together to create the balanced diet; a high protein low energy blend and a low energy high protein blend, then during a sudden period of heat stress, dietary interventions can be rapidly intervened to minimise losses. Diets containing high levels of protein are reported to have a higher heat increment than those containing high levels of carbohydrate or fat [35]. Thus, diets may be blended to reduce the heat increment by decreasing the high protein component of the diet during the hottest hours of the day. Another example of how precision feeding may be an important management tool for chicken-meat producers, is the ability for diets to be managed per farm to match the needs of each particular grower and their farm’s performance. Experienced producers or those situated in more favourable growing conditions may be able to generate good performance with slightly reduced dietary nutrient density, and thus reduced diet cost. However, it is currently impractical to pellet a different starter, grower, finisher and withdrawal diet based on performance for each individual chicken-meat producer. If protein and energy concentrate blends are provided, the ratio may be manipulated to suit each grower’s past performance to minimise the cost of production, and reduce bird variability across farms.

Another consideration during the implementation of precision nutrition for poultry is the way in which diets are formulated. Diets are typically formulated to a linear least-cost basis in order to reduce the cost of inputs. It is implied that formulating diets to least-cost will therefore generate the best profits for the business, but this is not necessarily the case [36,37]. The formulation of diets to maximise profits over the enterprise via the inclusion of meat yield, bird performance and economic data generates a more profitable outcome in an integrated operation. For example, Gonzalez-Alcorta et al [38] demonstrated that choosing dietary energy and protein levels which vary over the cost of inputs and outputs in a non-linear model can increase profit compared to those offered diets based on least-cost formulations set to traditional nutritional guidelines. The implementation of a precision nutrition regime would add to such max-profit approaches, as diets can be manipulated at any time in the growth cycle via adjusting dietary blends to manipulate energy and protein contents at an on farm level to meet fluctuating market requirements. Likewise, diets may be manipulated for certain producers to grow broilers for particular markets; for example, slow vs fast growing broilers.

Finally, as current farm systems are arranged to store three to four diets on farm, the possibility also exists for three to four feed blends to be implemented in order to increase the specificity by which the diets can be manipulated or treatments and additives included. For example, the ability to incorporate dietary additives on a short term basis within the feed is limited due to the complications in pelleting, transporting and storing over four to five diets for broiler production, and thus the incorporation of additives over a short term basis is restricted to water. This can be limiting as the additive must be water soluble. However, dietary additives may be able to be incorporated into a modified energy concentrate and then blended into the feed at the desired dose rate and time period to deliver the additive via the feed to the flock.

Thus, precision nutrition regimes for broiler production may not only provide advantages in attaining a more efficient nutrient utilisation, but may also benefit industry by maintaining the ability and fluidity to make dietary changes in energy and protein concentration at the day-to-day on farm level. Coupled with the increasing capacity of computing, big data, data management, and supply chain management within agriculture [38]; precision feeding technology and the concept of precision nutrition may be powerful tools for the future development of the Australian chicken-meat industry.

## CONCLUSION

Previous experiments exploring multi-phase feeding have supported the theory that increasing the number of diets to meet broiler nutritional requirements more precisely improves the efficiency of production. Thus, this has developed into the concept of precision nutrition; utilising modern precision feeding technologies to blend a new diet for each day of the production cycle. Recent precision nutrition research shows promise and the development of a precision nutrition regime which targets daily requirements by blending dietary components on farm is anticipated to improve the efficiency of production, reduce production cost and therefore improve sustainability of the industry. The Australian chicken-meat industry produces approximately 2.9 million tonnes of feed at a cost of $520/tonne or a total of $1.5 billion per annum. Therefore, the implementation of a precision nutrition regime has great capacity to reduce feed cost. There is also potential for precision feeding technology, coupled with precision nutrition regimes, to bring other management and economic benefits including increased fluidity to cope with sudden environmental or market changes and the ability to alter diets on a farm by farm level in a large, integrated operation. Integration into industry may require less investment than anticipated; producers may already possess the technology required to implement precision nutrition on farm via implementation of the blending capacity of some existing feed weighing systems. Nevertheless, there are few studies and further research to confirm the benefits of precision nutrition are required. Therefore, further study and exploration of the possibility of precision nutrition to enhance chicken-meat production is warranted. Precision nutrition regimes coupled with precision feeding technology may be the paradigm shift in feed formulation required to provide a future means to improve the efficiency and sustainability of the chicken-meat industry and meet the rising global demand for animal protein.
